# Resting energy expenditure and kidney disease: a narrative review

**DOI:** 10.3389/fnut.2025.1683191

**Published:** 2025-11-20

**Authors:** Zhuoxing Li, Sunhan Zhang, Xiang Xiao, Yun Sun

**Affiliations:** 1 School of Clinical Medicine, Chengdu Medical College, Chengdu, China; 2 Department of Nephrology, The First Affiliated Hospital of Chengdu Medical College, Chengdu, China

**Keywords:** kidney disease, resting energy expenditure, predictive equation, chronic kidney disease, acute kidney injury, renal replacement therapy

## Abstract

**Objectives:**

To explore the complex relationship between resting energy expenditure (REE) and kidney disease, and to synthesize evidence on REE assessment methods, influencing factors, and clinical implications for medical nutrition therapy (MNT).

**Background:**

Patients with kidney disease exhibit significant variations in metabolism and energy expenditure, increasing risks of comorbidities and adverse events. Accurate REE assessment is critical for precise energy intake planning and individualized MNT. However, current REE evaluation methods show inconsistencies, and factors driving REE changes (e.g., renal function, inflammation, comorbidities, medications) lack comprehensive analysis.

**Results:**

Significant discrepancies were identified across methodologies for assessing REE in kidney disease populations. Multiple disease-related factors—including renal function decline, inflammatory status, comorbidities, and pharmacotherapy—were found to dynamically alter REE patterns. While existing predictive equations offer clinical utility, they demonstrate notable limitations in applicability across diverse patient subgroups. Critically, addressing REE within personalized MNT significantly improves prognostic outcomes in this population.

**Conclusion:**

This review consolidates advances in REE-kidney disease research, establishes standardized assessment frameworks, and validates REE’s role in prognosis-focused MNT. It provides guidance for future studies and clinical practice, emphasizing REE optimization as essential for improving nutritional interventions in nephrology.

## 1 Introduction

Chronic kidney disease (CKD) is a significant public health issue globally. According to the latest data from the Global Burden of Disease (GBD) study, over 9.1% of the global population is currently affected by CKD. Including cases of acute kidney injury and other kidney diseases, the global prevalence of kidney disease exceeds 10% (1). Between 2010 and 2019, the prevalence increased by 16%. As the world gradually enters an aging population era and experiences a rise in metabolic diseases, it is foreseeable that more individuals will either develop kidney disease or be at risk of its development (2). It is estimated that global healthcare expenditures due to kidney diseases exceed hundreds of billions of dollars annually (3).

The kidneys are highly metabolic organs requiring substantial energy (4). A primary function is excreting nitrogenous waste, specifically urea produced by the liver from protein metabolism (5). When renal function declines, functional units are lost, obstructing urea excretion and elevating blood urea nitrogen (BUN). This disrupted protein metabolism is closely linked to abnormal energy expenditure, potentially becoming a key factor influencing patient energy consumption.

The maintenance of normal metabolic functions in the human body relies on a dynamic balance between energy intake and expenditure. Therefore, energy expenditure is often used to indirectly estimate the body’s energy requirements. There are three pathways for human energy expenditure: resting energy expenditure (REE), the thermic effect of food, and physical activity, with REE being the most significant component. REE refers to the energy required for an individual’s basic life activities while in a completely resting state. It is an important parameter for measuring individual energy needs and is significant for understanding human energy metabolism, formulating nutritional support plans, evaluating health status, and assessing energy balance in disease conditions (6). Although the kidneys accounted for only 0.4% of body weight, their energy consumption constituted approximately 7% of the total REE, second only to the heart (5). Furthermore, REE can be influenced by various factors, particularly in patients with kidney diseases, where the impact of these factors is more pronounced.

Meanwhile, kidney disease-related dietary factors continuously increase the metabolic burden on the kidneys, which is specifically manifested by an elevated glomerular filtration load and an increased tubular urea processing burden. Therefore, Medical Nutrition Therapy (MNT) plays a vital role in the management of kidney diseases. Energy intake is a key component of MNT, and relevant guidelines provide different recommendations for energy intake based on the type of kidney disease (7). Nevertheless, these recommendations cover a wide range, are relatively general, and fail to accurately assess an individual’s energy intake requirements (8).

Despite some advancements in research within this field in recent years, research related to the standardization of measurement methods, comprehensive analysis of influencing factors, practical long-term follow-up observations, the effectiveness of intervention measures, and healthcare professionals’ awareness remains fragmented and lacks systematic induction and integration. This limitation somewhat hinders the depth and progress of research. This study aims to systematically summarize and integrate research progress on REE in kidney disease patients, covering aspects such as characteristics, measurement methods, influencing factors, practical applications, and future prospects. The objective is to provide a more comprehensive and in-depth reference for research and practice in this field.

## 2 Composition and assessment methods of REE

### 2.1 Composition of REE

REE was the most stable component of total energy expenditure, accounting for 60 to 80% of the total energy expenditure (9). The assessment of REE clarifies the patient’s basal energy needs and also aids in the comprehensive evaluation of the patient’s total energy requirements, which is beneficial for healthcare professionals in formulating individualized energy intake plans. The physiological and pathological mechanisms of REE involve multiple aspects, including the energy demands of organs and tissues, metabolic processes, and the influence of body composition. Among the specific resting metabolic rates of seven organs and tissues in adults, the heart and kidneys exhibited the highest specific resting metabolic rates, which were twice that of the liver and brain (10, 11).

### 2.2 Measurement methods of REE

In practical applications, it is crucial to select appropriate measurement techniques based on research objectives, participant characteristics, and available equipment to ensure the accuracy and reliability of the results (Table 1).

**Table 1:** Measurement method/estimation approach for REE.

Measurement methods/ estimation approach		Advantages	Disadvantages
Direct calorimetry (12–14)		Principle of Direct Measurement: It directly measures the total heat dissipated by the body, providing a fundamental and accurate assessment of total energy expenditure. High Accuracy: Under controlled conditions, it is considered a very precise method for measuring total energy production.	Extremely Expensive: The equipment (the calorimeter chamber) is costly to build and maintain. Technically Complex: Requires a highly controlled and specialized laboratory environment. Impractical for Most Applications: The subject must remain inside the chamber for extended periods, severely limiting mobility and reflecting only activities that can be performed in a small, sealed room. It is not suitable for free-living conditions or routine clinical use. Does Not Provide Metabolic Substrate Information: Unlike indirect calorimetry, it cannot determine whether the body is burning carbohydrates, fats, or proteins for energy.
Indirect Calorimetry	Facemasks Ventilated hood systems (15–17)	Precise Measurement: The mask and headgear system accurately quantifies gas exchange throughout the respiratory cycle. Non-Invasive: These methods exert minimal impact on the subjects.	Stringent testing requirements: Participants must undergo a fasting period of no less than 2 h prior to the assessment and maintain a supine position for over 30 min during testing. The evaluation must be carried out in a quiet environment with temperature regulated between 20 and 25 degrees Celsius, and participants are expected to remain calm and attentive throughout the process. Elevated testing costs: The assessment necessitates specialized equipment, such as metabolic carts and cardiopulmonary function testing devices, along with disposable supplies.
	Whole-room calorimeters (18, 19) Respiration chambers (20)	High Accuracy: The system offers highly precise measurements of Resting Energy Expenditure (REE). Comprehensive Assessment: Beyond REE measurement, the metabolic energy chamber can analyze energy metabolism in relation to diverse diets, physical activities, and lifestyle factors, applicable in both healthy and disease-affected populations. Research Value: This tool is instrumental in the formulation and evaluation of nutritional support interventions, as well as in investigating the underlying mechanisms of metabolic diseases. Enhanced Comfort: The relatively spacious testing environment facilitates greater participant comfort during REE assessments, thereby minimizing the potential influences of stress and anxiety on measurement outcomes.	High Costs: The expenses related to equipment acquisition, operation, and maintenance are notably high, potentially restricting its adoption in specific research institutions or clinical settings. Currently, fewer than 40 laboratories globally are equipped with indirect calorimeters intended for human studies. Extensive Space Requirements: Metabolic energy chambers necessitate substantial spatial allocations, which may present challenges for laboratories or medical facilities constrained by limited space availability. Complex Operation: The operation and maintenance of metabolic energy chambers are relatively intricate, requiring the expertise of trained technicians for effective handling and upkeep.
	Handheld calorimetry (22–23)	Portability: Measurements can be performed in diverse environmental settings such as hospitals, clinics, homes, and research laboratories. Ease of Operation: In contrast to large-scale metabolic measurement devices, handheld calorimeters are user-friendly and typically do not necessitate complex setup or calibration procedures. Rapid Measurement: The assessment of REE can be accomplished in a brief timeframe. Cost-Effectiveness: Relative to high-end metabolic measurement devices, handheld calorimeters generally entail lower acquisition and maintenance costs, thereby enhancing their accessibility and applicability in clinical practice.	Measurement Accuracy: The accuracy of the measurements may not match that of more advanced metabolic measurement devices, potentially resulting in discrepancies between the recorded values and actual measures in certain instances. Environmental Influences: Measurement outcomes can exhibit instability due to the impact of environmental factors such as temperature and humidity. Individual Variance: The complexity of human metabolism may result in considerable differences in REE among individuals. Handheld calorimeters may not adequately account for these variations, leading to inaccuracies in measurement outcomes.
Doubly Labeled Water (26–27)		High Precision: The excretion rate of labeled isotopes in urine can be accurately tracked, enabling precise calculations of the body’s energy metabolism and expenditure. Non-Invasiveness: This method eliminates the need for subjects to perform specific physiological tasks or undergo invasive procedures; instead, subjects are simply required to consume a specified amount of doubly labeled water over a defined period. This approach minimizes discomfort and psychological stress for participants, thereby improving the acceptability of the measurements. Broad Applicability: This method is appropriate for large-scale epidemiological studies as well as long-term monitoring of energy metabolism. Real-Time Capability: Continuous collection and analysis of urine samples facilitate the real-time monitoring of individuals’ energy expenditure.	High Cost: The preparation and measurement of doubly labeled water necessitate expensive equipment and specialized techniques, thereby increasing the associated costs. Operational Complexity: The measurement process of the doubly labeled water method is relatively intricate, requiring precise control over the administration of isotopically labeled water and the timing of urine sample collection. This complexity mandates the involvement of skilled technicians for both operational execution and data analysis to ensure accuracy in measurement. Time Constraints: The evaluation of energy expenditure using the doubly labeled water method typically requires continuous urine sample collection over an extended period. Moreover, the transient nature of isotopically labeled water metabolism within the body imposes additional time limitations on the measurement process. Interfering Factors: Despite its high precision, the doubly labeled water method can be influenced by various interfering factors that may compromise measurement accuracy. Variables such as the subjects’ hydration status, dietary patterns, and levels of physical activity can significantly affect the excretion rate of the isotopic labels in urine, thereby impacting the reliability of the measurement outcomes.
Predictive equations	Predictive equation for the general population (30–33)	Convenience: The predictive equations offer a rapid and straightforward method for estimating REE without the necessity for intricate experimental measurements or specialized equipment. Cost-effectiveness: In comparison to the employment of specialized apparatus for REE measurement, predictive equations present notable economic advantages, particularly in resource-constrained environments or extensive population studies. Wide applicability: Typically derived from extensive population data, predictive equations encompass a diverse range of ages, genders, and body weights, rendering them appropriate for estimating REE within the general population. Non-invasiveness: The utilization of predictive equations for REE estimation obviates the need for invasive examinations or procedures, thus minimizing discomfort and psychological stress for participants.	Individual Differences: The inherent complexity of human metabolism results in considerable variations in REE among individuals. Existing predictive equations may inadequately reflect these differences. Equation Applicability: Various predictive equations may be appropriate for distinct populations or specific conditions. The inappropriate selection of equations or the failure to tailor them to the target population can result in biased estimations. Influencing Factors: These equations often neglect to consider fluctuations in individual metabolic rates, muscle mass, body fat percentages, the effects of diseases, and other relevant factors, leading to limitations in their accuracy. Update Requirements: Many commonly used equations were developed several decades ago, failing to correspond with the demographic characteristics of contemporary populations. Ongoing research and data validation are essential for their refinement.
	Predictive equation for patients with kidney disease (46, 128, 135–138)	Accuracy: The results obtained for patients with renal disease demonstrate significantly improved accuracy compared to those derived from the Harris-Benedict, WHO, or Schofield equations. Strong specificity: These equations consider the specific characteristics of patients with renal disease by incorporating targeted predictive factors such as C-reactive protein (CRP), hemoglobin A1C (A1C), serum creatinine (SCR), and albumin (ALB).	Individual Differences: While the predictive equations account for specific patient factors in kidney disease, significant variability in REE persists among different individuals with the condition. Applicability of Equations: Diverse predictive equations may be more appropriate for patients with kidney disease at various stages or under particular circumstances. Complexity of Influencing Factors: The REE in patients with kidney disease is influenced by a multitude of intricate factors, including disease severity, dialysis treatment, nutritional status, and medication usage. Consequently, these predictive equations may fail to encompass all relevant variables, leading to potential estimation errors.

#### 2.2.1 Direct calorimetry

Direct calorimetry is a method that directly measures the heat produced by individual cells during their activity (12). This approach is considered a highly accurate measurement technique. However, due to the requirement for complex monitoring tools and prolonged restrictions on individual activity (13), it has almost no practical applicability. As a result, direct calorimetry has largely been discontinued in real-world measurements (14).

#### 2.2.2 Indirect measurement method

Indirect calorimetry (IC) is the gold standard for measuring REE in clinical or laboratory settings (15–23). This method employs an indirect calorimeter to measure the amount of oxygen consumed and carbon dioxide produced by the body at rest. These measurements are then combined with respiratory quotients to estimate the individual’s glucose and fat consumption (18, 24) (Table 1).

#### 2.2.3 Doubly labeled water method

Although the Doubly Labeled Water (DLW) method is primarily used to measure total energy expenditure and is considered the gold standard for long-term assessment of daily energy expenditure (25), some studies have also explored its utilization for measuring REE (26). This method involves the oral administration of water containing stable isotopes to subjects, enabling the tracking of its metabolic processes within the body, and subsequently calculating energy expenditure indirectly (27) (Table 1).

#### 2.2.4 Predictive equations method

The predictive equations method is an estimation approach designed to estimate the energy expenditure of the human body at complete rest, utilizing demographic, body composition, and disease-related data. These equations allow for a rapid estimation of an individual’s REE (Table 1). Common predictive equations include the Harris-Benedict, FAO/WHO/UNU, Schofield, and Mifflin formulas (28–33), with specific equations detailed in Table 2.

**Table 2:** Predictive equations for REE of the general population.

Predictive equations	Variables	Predictive equation for resting energy expenditure	Creation time	Target population	Internal verification (R^2^)	External verification
Harris Benedict (30)	BW (kg), HT (cm), Age (year), Sex	M: 66.473 + 13.7516 × BW + 5.0033 × HT-6.755 × age F: 655.0955 + 9.5634 × BW + 1.8496 × HT-4.6756×age	1919	American	/	Yes
FAO/WHO/UNU (Weight and height) (31)	BW (kg), HT (cm), Age (year), Sex	Age: 18-30 M: 15.4 × BW-27 × HT + 717 F: 13.3 × BW + 334 × HT + 35 Age: 30 - 60 M: 11.3 × BW-16 × HT + 901 F: 8.7 × BW-25 × HT + 865 Age: ≥ 60 M: 8.8 × BW + 1128 × HT-1071 F: 9.2 × BW + 637 × HT-302	1985	Europeans	/	Yes
Schofield (Weight and height) (32)	BW (kg), HT (cm), Age (year), Sex	Age: 18-30 M: 0.063 × BW-0.042 × HT + 2.953 /4.184 × 1000 F: 0.057 × BW + 1.148 × HT + 0.411/4.184 × 1000	1985	White people in Europe	/	Yes
		Age: 30-60 M: 0.048 × BW-0.011 × HT + 3.67/4.184 × 1000 F: 0.034 × BW + 0.006 × HT + 3.53/4.184 × 1000 Age: ≥ 60 M: 0.038 × BW + 4.068 × HT - 3.491/4.184 × 1000 F: 0.033 × BW + 1.917 × HT + 0.074 /4.184 × 1000		and North America		
Mifflin et al. (33)	BW (kg), HT (cm), Age (year), Sex	M: 10 × BW + 6.25 × HT -5 × age + 5 F: 10 × BW + 6.25 × HT -5 × age-161	1990	American	0.71	Yes

Abbreviation: REE: Resting energy expenditure; BMI: body mass index; BW: body weigt; HT: height; FFM: fat free mass; FM: fat mass; M: male; F: female.

## 3 Energy metabolism characteristics

### 3.1 Intrinsic physiological regulatory factors

#### 3.1.1 Organ-specific metabolic rate

The kidneys are essential to the human body’s energy metabolism, functioning not only as excretory organs but also as endocrine organs involved in various metabolic processes. Renal cells are rich in mitochondria, which act as the cell’s ‘power plants’ generating adenosine triphosphate (ATP) to fuel cellular functions (34). The proximal renal tubule predominantly utilizes fatty acid oxidation for ATP production, serving as the central hub of renal energy metabolism (35). During periods of starvation or trauma, the kidneys can contribute up to 45% of the body’s energy through gluconeogenesis, a process critical for recovery and survival (36). While the renal cortex primarily depends on the oxidation of free fatty acids for energy, the renal medulla mainly relies on glycolysis to meet its energy demands (37). These metabolic processes not only supply energy for the kidneys themselves but also support the energetic needs of other organs in the body.

#### 3.1.2 Body composition

Body composition is a key determinant of REE, as metabolic rate is closely related to the proportion of metabolically active tissues in the body. Among various body components, fat-free mass (FFM) is the primary contributor to REE, accounting for approximately 60–70% of resting metabolic rate (25, 38, 39). In patients with CKD, one of the most notable alterations in body composition is the reduction in FFM, particularly in skeletal muscle mass. For this reason, FFM has become the most influential variable in constructing predictive formulas for REE (40).

However, the relationship between body composition and REE in CKD patients is more complex than simple muscle loss. Some patients in the early stages of CKD may be overweight or obese. Since FFM is positively correlated with body weight, individuals with obesity and higher FFM levels may also exhibit an increase in REE (41). This can lead to an elevated overall REE in unadjusted models in studies that include patients with high BMI (42), which masks the negative impact of pure muscle atrophy on REE and highlights the importance of distinguishing between FFM and adipose tissue in such analyses.

Furthermore, the assessment itself poses challenges in the CKD population. Fluid overload, commonly seen in advanced CKD and ESRD, can interfere with body composition measurements based on bioelectrical impedance analysis (BIA) (43). Excessive extracellular fluid alters BIA measurements, making it difficult to accurately differentiate between FFM and adipose tissue, thereby affecting the interpretation of the true relationship between REE and body composition. This edema may also have minor metabolic effects.

#### 3.1.3 Demographic factors

Age is a fundamental factor influencing REE, primarily due to reduced muscle mass and decreased cellular metabolic activity. This phenomenon is particularly pronounced in kidney disease patients, as the process is significantly accelerated (44, 45). The combined effects of uremia, chronic inflammation, metabolic acidosis, and physical inactivity create a catabolic environment that exacerbates age-related muscle loss. Consequently, elderly CKD patients may exhibit abnormally low REE relative to their body weight. In multiple multivariate regression models analyzing REE in kidney disease patients, the regression coefficient for age remains consistently negative (46). This indicates that among patients with the same body weight and gender, older individuals tend to have a relatively lower basal metabolic rate. The impact of age on REE becomes more variable in hemodialysis patients. Some studies suggest that elderly hemodialysis patients may demonstrate even lower REE due to more severe loss of FFM (47).

Gender influences REE primarily through muscle mass and hormone levels. As men generally have higher muscle mass, this gender difference persists in patients with kidney disease, potentially due to men’s greater muscle reserves and the role of testosterone in maintaining metabolism (48). However, female CKD patients may experience more pronounced declines in REE due to accelerated muscle loss associated with decreased estrogen levels. In patients with CKD stages 3–5, serum leptin levels were significantly positively associated with REE in men rather than in women, further suggesting that gender may influence energy metabolism through differences in hormones or fat distribution (49).

### 3.2 Pathophysiological regulatory factors

**Figure 1:**
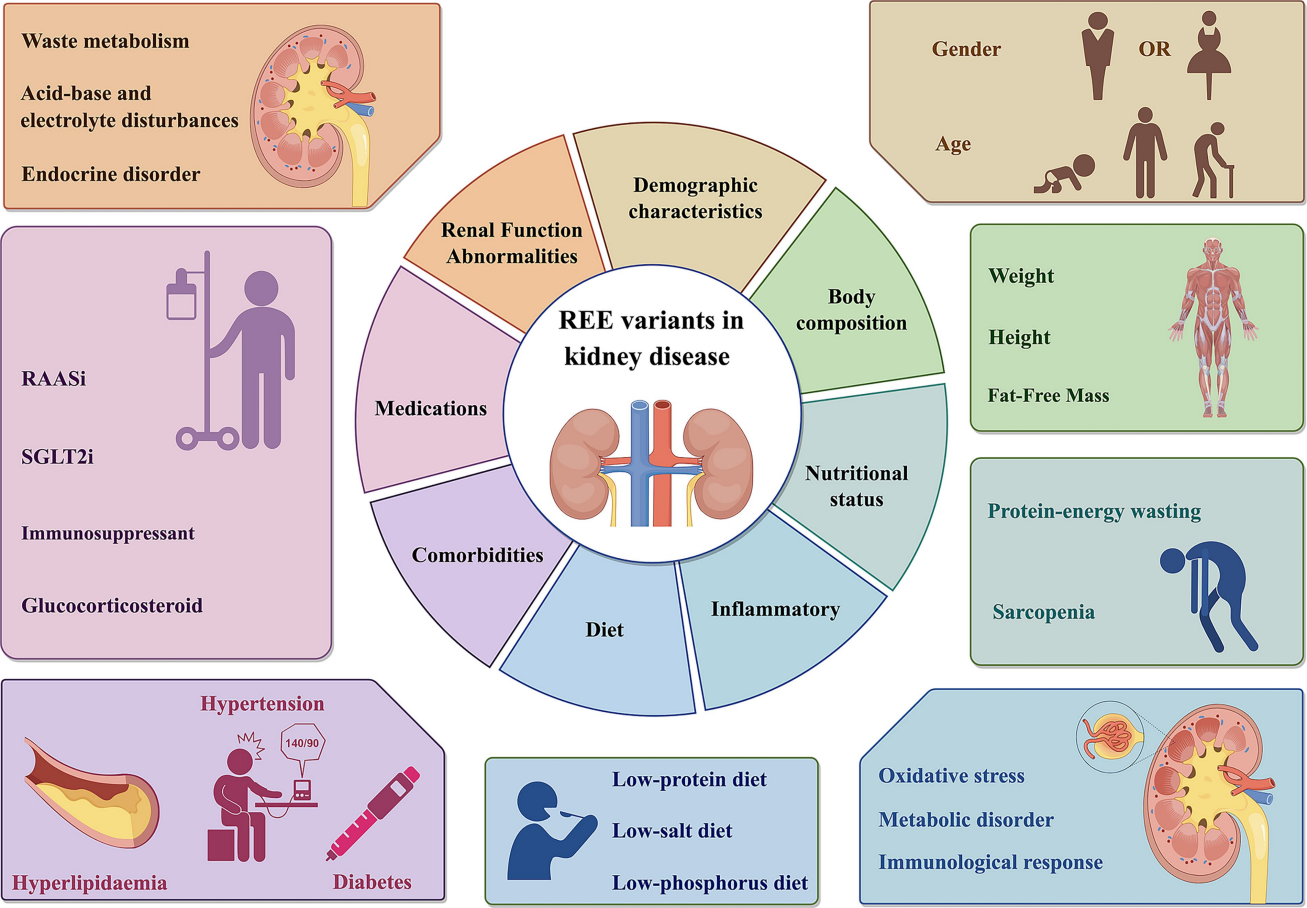
REE variants in kidney disease. The REE of patients with renal diseases is influenced by multiple factors, including body composition, demographic characteristics, renal function abnormalities, nutritional status, inflammatory, comorbidities, medications. REE, Resting energy expenditure; SGLT2i, Sodium-Glucose Co-Transporter 2 Inhibitor; RASSi, Renin-Angiotensin-Aldosterone System Inhibitors. This figure was created using Figdraw.com.

The changes in REE in patients with kidney disease are regulated by multiple pathophysiological mechanisms. Whether REE increases or decreases often depends on the dominant pathological state and clinical characteristics of the patient at different stages of the disease. Specifically, during periods of uremic toxin accumulation or malnutrition, REE tends to suppressed; whereas, in conditions such as active inflammation, metabolic acidosis, insulin resistance, or hypercatabolic states, REE may be increase. Therefore, the final change in REE reflects a dynamic balance between mechanisms that promote energy expenditure and those that suppress it. In clinical practice, a comprehensive assessment—incorporating the patient’s specific disease course, metabolic status, nutritional indicators, and inflammatory levels—remains essential (Figure 1).

#### 3.2.1 Renal function abnormalities

Renal function abnormalities markedly influence REE. From the perspective of renal clearance of metabolic waste, renal failure results in the accumulation of metabolites such as BUN and creatinine (Cr) in the body. These substances disrupt normal cellular metabolism and may exacerbate oxidative stress by impairing mitochondrial function, thereby hindering ATP synthesis and utilization (50).

Moreover, disturbances in the metabolism of electrolytes, such as water, sodium, potassium, calcium, and phosphorus, as well as imbalances in acid–base homeostasis, directly impact essential physiological functions of cells, including membrane potential and ion channel activity. This disruption subsequently affects energy metabolic pathways (51). Abnormal function of the sodium-potassium pump can lead to ionic imbalances across cellular membranes, impairing ATP generation and utilization. Given that the sodium-potassium pump was crucial for maintaining resting potentials and that Na+/K + ATPase accounted for approximately 25% of cellular energy expenditure (52), any dysfunction can result in a significant decline in the absolute value of REE.

In addition, renal failure may cause dysregulation of hormones such as erythropoietin (EPO) and 1,25-dihydroxyvitamin D3 (53), both of which indirectly affect REE. Animal studies demonstrated that EPO could influence activity levels, total oxygen consumption, and the respiratory quotient (RQ) (54). In a study that involved healthy young males receiving recombinant human EPO treatment, an increase in REE was observed, alongside a trend toward enhanced fat oxidation (55). Furthermore, a deficiency in 1,25(OH)2D3 can disrupt calcium-phosphorus metabolism, leading to skeletal muscle weakness, which further diminishes REE (56).

#### 3.2.2 Nutritional status

The kidney is a core organ responsible for maintaining the body’s metabolic balance. When renal function is impaired, it leads to a series of metabolic disorders, making patients highly susceptible to malnutrition. This condition is not merely an issue of insufficient nutrient intake but rather a complex state of protein-energy wasting (PEW) (57). PEW is a common form of malnutrition in CKD patients, characterized by the progressive depletion of protein and energy reserves in the body (58). PEW can occur across all BMI levels, including in individuals with obesity. A significant proportion of the CKD and ESRD population is overweight or obese. Although obesity is generally associated with a higher absolute REE due to increased body mass, obesity in the context of CKD is often “sarcopenic obesity,” with approximately 10.8% of CKD patients suffering from this condition (59). It is characterized by an abnormally increased proportion of adipose tissue, coupled with a relative or absolute reduction in metabolically active lean body mass. Since the metabolic rate of adipose tissue is much lower than that of muscle tissue, this imbalance in body composition can lead to a reduction in both absolute and relative overall REE (60). Although fat-related inflammation may contribute to some increase in metabolism, it is generally insufficient to fully compensate for the reduced energy expenditure caused by muscle loss.

In healthy individuals, plasma amino acids are finely regulated during fasting to maintain a dynamic equilibrium, thereby supporting protein synthesis and energy supply (61). However, in patients with kidney disease, severe renal impairment leads to abnormalities in the fasting plasma amino acid profile: on one hand, levels of certain essential amino acids decrease, while on the other hand, the accumulation of nitrogenous waste products causes systemic metabolic disturbances (62). This not only directly inhibits protein synthesis but also indirectly affects resting energy expenditure by altering substrate utilization. At the same time, the buildup of uremic toxins suppresses or abnormally activates key metabolic enzymes, exacerbating protein breakdown and leading to muscle loss and weight reduction (63). Although a low-protein diet can alleviate the nitrogen load and reduce renal burden, insufficient protein intake may worsen the patient’s nutritional status (64). Furthermore, these patients often experience a hypermetabolic state accompanied by insulin resistance, inflammatory responses, metabolic acidosis, and other issues, all of which collectively promote protein degradation. This inhibition of protein synthesis and acceleration of protein breakdown, driven by amino acid metabolic dysregulation, collectively alters the body’s metabolic rate and energy expenditure patterns, serving as an important pathophysiological basis for the abnormal increase in resting energy expenditure in patients with kidney disease.

#### 3.2.3 Inflammation

Patients with kidney diseases often exhibit a microinflammatory state, characterized by elevated levels of interleukin-6 (IL-6), tumor necrosis factor-alpha (TNF-α), and C-reactive protein (CRP) (65). This microinflammatory state significantly influences REE through various mechanisms.

##### 3.2.3.1 Metabolic disorders leading to enhanced inflammatory response

Metabolic disorders in individuals with kidney diseases may activate the NF-κB signaling pathway, facilitating the release of inflammatory mediators such as IL-6 and TNF-*α*. This activation adversely affects fat and glucose metabolism, thereby increasing REE (66). IL-6 exacerbates lipolysis, resulting in lipid metabolic disturbances and insulin resistance (67). Concurrently, TNF-α diminishes *β*-oxidation in adipose tissue and promotes lipogenesis via the NF-κB pathway, which hampers the adipose tissue’s capacity to fulfill the ATP demands imposed by CKD, ultimately redirecting metabolism toward anaerobic pathways. Although anaerobic metabolism can considerably elevate REE in cancer patients, its specific effects on REE in kidney disease patients warrant further exploration. It should be noted that during the late stages of CKD, REE may decline due to muscle wasting and a generalized reduction in metabolic rate (68).

##### 3.2.3.2 Immune response mediating inflammatory response

During inflammation, the body’s immune response is heightened, necessitating significant energy and protein consumption to support the proliferation and differentiation of immune cells, which consequently leads to an increased REE. Furthermore, inflammation activates the monocyte–macrophage system, resulting in an elevated production of pro-inflammatory cytokines that exacerbate malnutrition and further elevate REE (69). In the context of CKD, a decline in glomerular filtration rate (GFR) contributes to the accumulation of toxins within the body, which may activate the immune system and promote inflammatory responses (70).

##### 3.2.3.3 Oxidative stress leading to inflammatory injury

Oxidative stress is frequently observed in patients with kidney disease, and its various metabolic mechanisms can increase REE (71). Oxidative stress can result in mitochondrial dysfunction alongside an increased production of reactive oxygen species (ROS) (72). ROS can inflict damage on cell membranes, proteins, and DNA, leading to cellular dysfunction (73). Additionally, the elevated ROS levels found in the blood of end-stage renal disease (ESRD) patients foster the release of inflammatory factors. Such inflammatory responses activate mitochondria and NADPH, thereby amplifying ROS production and establishing a vicious cycle that exacerbates both inflammation and oxidative stress levels (74, 75). Furthermore, oxidative stress can trigger the activation of the nucleotide-binding domain (NBD), leucine-rich repeat (LRR), and pyrin domain (PYD)-containing protein 3 (NLRP3) inflammasome, facilitating the release of inflammatory mediators via the NF-κB signaling pathway. This activation results in the secretion of pro-inflammatory cytokines such as IL-1β and IL-18, further aggravating tissue damage (76, 77). Oxidative stress can also promote the dissociation of thioredoxin-interacting protein (TXNIP) from endogenous antioxidants, allowing it to engage with the NLRP3 inflammasome and subsequently activate the inflammatory cascade (78, 79).

#### 3.2.4 Comorbidities (such as diabetes, hypertension, and hyperlipidemia)

Patients with kidney diseases often have multiple comorbidities, such as diabetes, hypertension, and hyperlipidemia, which significantly affect the REE of patients. The REE of diabetic patients at different levels of glycated hemoglobin is significantly higher than that of healthy controls (80). This finding underscores the considerable alterations in resting energy metabolism that occur in the context of diabetes. In animal studies, diabetes has been shown to provoke a remarkable 360% increase in renal glucose release in rats, and a 300% increase in humans, with concomitant rises in REE (81, 82). The kidney plays a crucial role in regulating insulin sensitivity. Therefore, insulin resistance, which is common in diabetic patients, is further exacerbated when combined with kidney disease (83). Studies have shown that in individuals with insulin resistance, energy expenditure for gluconeogenesis can account for 22 to 39% of their REE, significantly higher than in those with normal body weight (84). This abnormally elevated gluconeogenic activity is likely to increase overall resting energy expenditure levels (85). Additionally, hyperglycemia can inflict damage on endothelial and smooth muscle cells, leading to microvascular complications that may indirectly influence REE by altering energy metabolism pathways (86).

Hypertension not only places a significant burden on renal function but also markedly increases REE in patients with kidney disease. It can impair the kidneys’ filtration capabilities, thereby reducing the effectiveness of waste elimination, which may lead to the onset of PEW (87). Additionally, hypertension can alter renal hemodynamics, creating hypoxic and ischemic conditions in renal tissues that adversely affect renal oxygen metabolism and mitochondrial bioenergetics (88). For example, compared to Wistar-Kyoto rats with normal blood pressure, spontaneously hypertensive rat (SHR) models demonstrate a substantial reduction in renal medullary blood flow and a marked decrease in oxygen partial pressure, resulting in a significant decline in oxygen utilization efficiency (89). This hypoxic environment may precipitate a cascade of metabolic disorders (90), causing REE in patients with CKD to exceed that observed in healthy individuals.

In individuals with renal insufficiency, hyperlipidemia can aggravate oxidative stress and inflammatory responses, leading to disturbances in energy metabolism. Under hyperlipidemic conditions, AMP-activated protein kinase (AMPK) is activated, inhibiting the activity of fatty acid synthesis-related enzymes such as SREBP-1 and SREBP-2, while concurrently upregulating the expression of key fatty acid oxidation enzymes including ACOX1, CPT-1, and CPT-2 (91). Although this mechanism mitigates lipid accumulation, it concurrently affects overall energy balance (92). The excessive lipid accumulation in the kidneys due to hyperlipidemia may induce lipotoxicity, activating multiple signaling pathways that drive oxidative stress, inflammation, fibrosis, endoplasmic reticulum stress, and apoptosis, further exacerbating abnormal energy metabolism (93).

### 3.3 Diet

Among the many factors affecting the REE of patients with kidney disease, disease-specific dietary regimens play a crucial role. A low-protein diet directly reduces the high thermic effect of food associated with protein digestion and absorption, but more importantly, it may induce a state of PEW. When exogenous high-quality protein intake is insufficient, the body is forced to break down its own skeletal muscle for gluconeogenesis. This endogenous substrate conversion process is energy-inefficient and may abnormally increase REE (94). However, the long-term loss of muscle mass reduces the body’s largest energy-consuming tissue, ultimately leading to a decline in the basal metabolic rate (95). The direct impact of a low-salt diet on the REE of kidney disease patients has rarely been demonstrated by research. Nonetheless, it reduces blood volume and blood pressure, effectively alleviating the long-term burden on the heart and minimizing unnecessary energy expenditure for maintaining cardiovascular function (96). Additionally, controlling salt intake helps suppress the overactivated sympathetic nervous system and the renin-angiotensin-aldosterone system, thereby reducing the hypermetabolic state induced by these systems (97). Phosphorus is a core component of ATP, the energy currency of cells, and its metabolic disturbances directly affect the efficiency of mitochondrial oxidative phosphorylation. A low-phosphorus diet is often combined with a low-protein diet, further restricting food choices and total caloric intake, thereby exacerbating the risk of energy imbalance (98).

### 3.4 Therapeutic medications

Commonly prescribed glucocorticoids, such as methylprednisolone and prednisone, have been shown to elevate REE in patients with kidney diseases by modulating calcium cycling in skeletal muscle (99, 100). Similarly, patients receiving immunosuppressants, including cyclophosphamide and cyclosporine, often experience an increase in REE exceeding 10% (101). Antidiabetic medications used by individuals with diabetic nephropathy can influence glucose and lipid metabolism, leading to insulin resistance, altered fat distribution, and sodium retention (102–104). In overweight or obese women with hypertension, angiotensin-converting enzyme inhibitors (ACEI) and angiotensin receptor blockers (ARBs) demonstrated a reduction in REE (105); comparable findings have been observed in animal studies (106, 107). However, further investigation is necessary to establish a definitive association between renin-angiotensin system inhibitors (RASSi) and REE in patients with kidney disorders. Sodium-Glucose Co-Transporter 2 Inhibitor (SGLT2i) were known to increase REE through the activation of the AMPK pathway, concomitant with alterations in lipoprotein and leptin expression (108). Conversely, some studies reported that energy expenditure in patients with type 2 diabetes (T2D) remained unchanged with the use of dapagliflozin or empagliflozin (109, 110). This phenomenon may be attributed to a sustained negative energy balance and subsequent weight loss, which can instigate compensatory hyperphagia, resulting in increased energy intake (111). Therefore, the effects of SGLT2i on human REE necessitate further investigation.

Furthermore, the effects of medications on kidney function can be evaluated through their influence on energy metabolism (112). Certain drugs, particularly non-steroidal anti-inflammatory drugs (NSAIDs) and aminoglycosides, have been definitively linked to renal toxicity, potentially causing AKI through alterations in renal tubular secretion or changes in glomerular filtration rate (113, 114). The metabolic disturbances or nephrotoxic effects induced by these agents may manifest as alterations in the patient’s energy metabolism patterns.

In conclusion, effective management strategies for patients with kidney diseases should include comprehensive oversight of estimated Glomerular Filtration Rate (eGFR), acid–base and electrolyte balance, inflammation, nutritional status, blood glucose levels, blood pressure, blood lipid profiles, and pharmacological interventions to ensure the stable maintenance of REE.

## 4 Energy expenditure in various renal diseases

### 4.1 AKl

#### 4.1.1 Characteristics of REE in AKI

The influence of AKI on REE is characterized by a range of metabolic alterations, diminished nutrient utilization, and adaptations in overall physiological condition subsequent to renal impairment. Specifically, AKI patients exhibit enhanced insulin resistance due to renal glycogen depletion and decreased insulin and glucagon clearance, resulting in hyperglycemia and elevated insulin levels, which serve as critical indicators of disease severity (115). This distinctive metabolic profile necessitates that REE in AKI patients not only supports essential physiological functions but also meets the increased energy demands associated with metabolic dysregulation.

Furthermore, the REE of AKI patients is influenced by Continuous Renal Replacement Therapy (CRRT). During CRRT, the loss of calories and small to medium-sized nutrients, including glucose, amino acids, select vitamins, and trace elements, becomes significantly pronounced (116, 117). The extent of these losses was closely associated with CRRT parameters such as blood flow rate, replacement volume, temperature, dilution methods, and the type of dialysis employed (118). However, Goes et al. (119) compared AKI patients who underwent Conventional Hemodialysis (CHD), Extended Hemodialysis (EHD), and High-Volume Peritoneal Dialysis (HVPD) and found no notable differences in REE post-dialysis across the various modalities. Thus, the impact of dialysis treatment on the REE of AKI patients remains a contentious issue within the field.

#### 4.1.2 Prediction methods for REE in AKI

In the nutritional management of patients with AKI, it is essential to comprehensively consider the changes in REE and the various metabolic abnormalities mentioned above that impact REE. Although AKI patients have an urgent need for accurate assessment of energy and nutritional requirements, the predictive efficacy of most classical prediction equations is suboptimal (120), and currently, no REE prediction equations suitable for clinical application have been developed. Regarding the assessment of REE in AKI patients, only Ponce D et al. constructed a predictive model using machine learning (121). However, this model lacks rigorous external validation and sufficient evidence to confirm its effectiveness in practical clinical settings.

### 4.2 Non-dialyzed chronic kidney disease

#### 4.2.1 Characteristics of REE in ND-CKD patients

Patients with CKD endure a progressive decline in renal function, leading to inadequate excretion of metabolic waste products and toxins. This deterioration precipitates systemic metabolic abnormalities that directly or indirectly affect REE levels. Compared to healthy individuals, the REE in CKD patients is approximately 123 kcal/d lower (122).

In the early stages of CKD (Stages 1–3), REE primarily shows a trend of being relatively normal or mildly elevated (123). Even in the early stages, some patients may already have a state of microinflammation, with mildly elevated inflammatory cytokines, which may tend to increase REE. However, during this period, factors leading to decreased REE are generally not yet apparent.

In the late stages of non-dialysis dependent CKD, patients often experience reduced muscle mass and changes in fat distribution, a condition known as ‘renal disease-related sarcopenia’ (59). Given that muscle was the primary tissue responsible for generating REE, a decrease in muscle mass leads to a decline in REE (124). Concurrently, patients with late-stage CKD are more significantly affected by metabolic syndrome (MetS) and hormonal regulation. Given that MetS is associated with cellular inflammation and enhanced immune cell activation, REE in patients with MetS tends to be relatively high (125). The kidneys function as metabolic and excretory organs for various endocrine hormones, such as parathyroid hormone, vitamin D, and erythropoietin. In CKD patients, the metabolism and excretion of these hormones may be impaired, leading to abnormal hormone levels. For example, elevated parathyroid hormone levels can stimulate the catabolism of skeletal muscle, thereby increasing REE (126), while a deficiency of vitamin D may trigger muscle weakness and atrophy, subsequently reducing REE (127).

#### 4.2.2 REE prediction equations in ND-CKD patients

Numerous research teams have developed equations to predict REE for ND-CKD patients. Fernandes et al. introduced three novel REE prediction equations tailored for ND-CKD patients (128). These equations leveraged variables associated with the patients’ body composition, including weight, height, and fat-free mass. Additionally, Xu et al. (46) formulated and validated a new REE prediction equation specifically for ND-CKD patients, which incorporated fundamental patient data such as gender, age, weight, and the presence of diabetes. Comparisons with REE measurements obtained via indirect calorimetry indicated that the accuracy of these equations within the validation cohort was satisfactory. (Table 3).

**Table 3:** Predictive equations for REE in patients with kidney disease.

Predictive equations	Predictive equation for REE	Applicable population	Creation time	Target population	Internal verification (R^2^)	Validation status
Fernandes REE (128)	(1) Use BW calculation: M = 1033 + 7.4 × BW - 3.3 × age + 2.1 ×eGFR + 26 × (1 if DM; 0 if non-DM) F = 854 + 7.4 × BW - 3.3 × age + 2.1 × eGFR + 26 × (1 if DM; 0 if non-DM)	ND-CKD	2021	Brazilian	0.424	Yes
	(2) Use FFM (by anthropometry) calculation: M = 733.1 + 14.07 × FFM.ant - 2×age + 2.5×eGFR + 140.7 × (1 if DM; 0 if non-DM) F = 678.3 + 14.07 × FFM.ant - 2×age + 2.5×eGFR + 140.7 × (1 if DM; 0 if non-DM)				0.449	
	(3) Use FFM (by bioelectrical impedance) calculation: M = 575.3 + 17.1 × FFM.BIA - 2.7 × age + 1.3 × eGFR - 152.3 × (1 if DM; 0 if non-DM) F = 668 + 17.1 × FFM.BIA - 2.7 × age + 1.3 × eGFR - 152.3× (1 if DM; 0 if non-DM)				0.45	
Xu REE (46)	M = 751.5 - (1 if DM; 0 if non-DM) × 51.6-4.7 × age + 13.1 × BW F = 645.5 - (1 if DM; 0 if non - DM) × 51.6 - 4.7 × age + 13.1 × BW	ND-CKD	2021	Chinese	0.779	Yes
Vilar REE (135)	Age:<65 M = 0.011 × HT^2.023^ + 83.573 × BW^0.6291^ + 68.171 F = 0.011 × HT^2.023^ + 83.573 × BW^0.6291^ Age: ≥ 65 M = -2.497 × age + 0.011 × HT^2.023^ + 83.573 × BW^0.6291^ + 68.171 F = -2.497 × age + 0.011 × HT^2.023^ + 83.573 × BW^0.6291^	MHD	2014	Caucasian	0.663	Yes
The MHD Equation (MHDE-REE) (136)	Base Model (MHDE-Base): REE = 409.44 + 15.35 × FFM-6.64 × age + 4.12 × (Sex:1 if M; 0 if F) + 193.82 × ALB + 4.59 × CRP	MHD	2014	African American	0.489	Yes
	Best Predictive Model (MHDE-LBM): REE = 404.58 +15.44 × FFM - 6.62 × age + 194.45 × ALB + 4.58 × CRP				0.489	
	(3)Model with the Best Clinical Utility when CRP is Available (MHDE-CRP): REE = 808.20 + 4.96 × BW-8.66 × age + 1192.52 × ALB + 4.38 × CPR + 140.14 × (Sex:1 if M; 0 if F)				0.460	
	(4)Model with the Best Clinical Utility when LBM and CRP are Unavailable (MHDE-Creatinine): REE = 750.15 + 5.38 × BW-8.207 × age + 188.98 × albumin + 137.94 × (Sex:1 if M; 0 if F) + 6.68 × CR				0.451	
Byham-Gray REE (137)	(1)Maintenance Hemodialysis Equation (MHDE-CRP 2016): M = 1027.8 -5.19×age +9.67 × BW +2.71 × CRP F = 820.47 -5.19×age + 9.67 × BW +2.71 × CRP	MHD	2018	African American	0.67	Yes
	(2)Maintenance Hemodialysis Equation (MHDE-A1C 2016): M = 1128.06 -4.62×age + 10.33×BW -21.79×A1C F = 886.7 -4.62 × age + 10.33 × BW -21.79 × A1C				0.66	
	(3)Maintenance Hemodialysis Equation (MHDE-SCr 2016): M = 1024.41-4.90 × age +10.21 × BW-3.25 × SCr F = 802.00-4.90×age +10.21×BW-3.25×SCr				0.66	
Fernandes REE (138)	(1) Use BW calculation: M = 957.02 - 8.08 × age + 11.07 × BW+ 136.4 F = 957.02 – 8.08 × age + 11.07 × BW	MHD	2019	Brazilian	0.515	Yes
	(1) Use FFM calculation: M = 615.95-4.8 × age+20.6 × FFM F = 624.6 -4.8 × age+20.6 × FFM				0.512	

Abbreviation: REE: Resting energy expenditure; BMI: body mass index; BW: body weigt; HT: height; FFM: fat free mass; FM: fat mass; M: male; F: female; CRP: c-reactive protein; A1C: glycosylated hemoglobin; SCr: serum creatinine; CR: creatinine; NDD-CKD: non-dialyzed with.

#### 4.2.3 The relationship between REE and prognosis in ND-CKD patients

A notable negative correlation was identified between REE and eGFR, suggesting that elevated REE levels were associated with an increased risk of ND-CKD progression (129). An analytical study examining patients with Diabetic Kidney Disease (DKD) revealed that individuals with lower REE levels faced a 6.08-fold increased risk of advancing to ESRD compared to those with higher REE levels (130). Thus, the influence of REE on various stages of CKD development may exhibit variability or be specific to certain populations.

### 4.3 Different kidney replacement therapy methods for ESRD

#### 4.3.1 Dialysis

##### 4.3.1.1 Characteristics of REE in hemodialysis patients

During HD, metabolic waste and toxins are effectively removed from the patient’s body, thereby enhancing their overall metabolic status. However, HD can also provoke acute physiological responses, such as hypotension and muscle cramps, which may result in transient increases in REE. Notably, during the initial phase of HD, the body may experience a degree of REE elevation as it acclimates to the altered metabolic environment. Over time, as the HD process continues and the patient adapts, REE levels typically stabilize. Consequently, some studies observed that there was no significant difference in REE between HD patients and healthy adults (131). Additionally, CRRT may elicit inflammatory responses due to interactions between blood and exogenous substances, resulting in biocompatibility issues that further elevate REE (132).

##### 4.3.1.2 Characteristics of REE in peritoneal dialysis patients

Patients undergoing PD encounter various complications, including peritoneal infections and protein malnutrition. The dialysis process can lead to protein loss from the dialysis solution, which subsequently lowers plasma ghrelin levels, diminishes appetite, and further aggravates malnutrition, thereby increasing REE (133). Additionally, the presence of fluid in the abdominal cavity may interfere with body composition assessments, resulting in inaccurate REE measurements (134).

##### 4.3.1.3 REE assessment equations for dialysis patients

For the HD population, existing research has established targeted equations that more accurately reflect the patients’ disease stages and clinical characteristics (Table 3). However, each methodology has inherent limitations and must take into account specific patient circumstances, including comorbidities, when determining the most suitable assessment approach.

Vilar et al. (135) developed a REE equation utilizing multiple linear regression, incorporating variables such as height, weight, sex, and age. In 2014, Byham-Gray established REE equations specifically for patients undergoing MHD, addressing limitations encountered in clinical settings. However, due to issues such as sampling bias, small sample sizes, and incomplete data regarding potential predictive factors, the results warranted cautious interpretation (136). In 2018, the research team refined the equations by adding variables, including weight, age, sex, and C-reactive protein (CRP) (137). The revised model significantly enhanced clinical predictability by involving patients with DKD. Nevertheless, approximately 40% of the actual REE variance remained unexplained, indicating the necessity for further refinement in prospective studies. Fernandes et al. proposed new estimation equations incorporating fat-free mass (FFM), weight, sex, and age, while also evaluating the accuracy of the REE dialysis equations developed by Vilar and Byham-Gray (138). They found that the Vilar equation tended to overestimate REE, and both equations displayed systematic and proportional biases. Subsequent investigations revealed that the MHDE REE, Vilar REE, and Fernandes REE equations performed inadequately when tested in other populations (22, 139–141). Additionally, Bailey et al. constructed a machine learning model with 167 samples from the Rutgers Nutrition and Kidney Database (RNKD), achieving an impressive accuracy of 91.2% with the best-performing model, Support Vector Regression (SVR) (142).

Among these equations, only a few have undergone empirical validation. Most of these studies have focused on specific clinical subgroups and involved relatively limited sample sizes. Although validation results indicate that these equations can reasonably predict REE within clinically acceptable error margins in the selected populations, the limited number of studies makes it difficult to draw strong conclusions regarding their generalizability. Another critical issue lies in the potential discrepancies between the population characteristics used during equation development, such as age distribution, gender ratio, baseline health status, type and severity of kidney disease, and those of the populations used in subsequent validation studies. Future research should systematically validate existing equations across diverse patient subgroups within large, multicenter cohorts of kidney disease patients and report their accuracy and bias in different populations.

##### 4.3.1.4 The relationship between REE and prognosis in dialysis patients

Research indicated a correlation between elevated REE and increased mortality, specifically cardiovascular death rates, in patients undergoing Continuous Ambulatory Peritoneal Dialysis (CAPD). This association was partly attributed to the interdependence of REE with residual kidney function, cardiovascular disease, inflammation, and malnutrition in this patient population (115). Furthermore, an increase in REE is a critical factor contributing to malnutrition and muscle wasting among peritoneal dialysis patients (143).

#### 4.3.2 Kidney transplantation

##### 4.3.2.1 Characteristics of REE in kidney transplantation

Kidney transplantation is a crucial intervention for ESRD and significantly influences patients’ REE. The surgical procedure itself is inherently traumatic, triggering the body’s stress response and initiating a catabolic metabolic state (144). During the early postoperative period, energy demands are elevated due to surgical stress and increased catabolism. Consequently, REE typically increases to meet physiological requirements for wound healing, infection resistance, and other bodily functions (145).

Additionally, the use of immunosuppressants plays a critical role in influencing REE following kidney transplantation. These medications not only attenuate immune function but also disrupt metabolic processes by promoting gluconeogenesis and diminishing peripheral glucose utilization, ultimately leading to hyperglycemia and dyslipidemia (101, 146, 147). Such metabolic alterations can affect REE and disrupt the equilibrium between energy demands and expenditures.

## 5 Application of REE in the diagnosis and management of kidney disease

### 5.1 Evaluation of REE to facilitate nutritional intervention plans

The primary aim of nutritional support in patients with kidney disease is to supply adequate calories and protein to sustain normal physiological functions, enhance recovery, and minimize the risk of complications.

Patients with kidney disease frequently experience metabolic disorders, such as PEW and MetS, which can deteriorate alongside declining renal function, significantly affecting patient prognosis. The Kidney Disease Outcomes Quality Initiative (KDOQI) recommends a daily caloric intake of 25–35 kcal/kg for individuals with CKD to satisfy energy requirements (7). However, this general guideline is challenging to tailor to individual patients, complicating precise energy intake recommendations. Therefore, adjusting nutritional support plans based on REE in the management of kidney disease ensures that patients receive adequate energy while mitigating the risks associated with excessive caloric intake.

### 5.2 Evaluation of REE and its role in predicting malnutrition risk

Patients with kidney disease often experience metabolic abnormalities and nutritional deficiencies, which not only affect their quality of life but may also accelerate the progression of the disease. By regularly monitoring REE and correlating it with other indicators, the accuracy and sensitivity of disease diagnosis can be improved, allowing for timely detection of abnormal shifts in the patient’s metabolic state. Meanwhile, healthcare professionals can utilize the dynamic monitoring results of REE to formulate corresponding nutritional support plans, implementing nutritional interventions specifically for patients with kidney diseases. For patients with lower REE, it is advisable to moderately increase protein and caloric intake to maintain their nutritional status and immunity; conversely, for patients with higher REE, greater attention should be given to the balance and adequacy of their diet to prevent excessive energy intake that could lead to obesity and other complications (133).

A dynamic perspective can reveal the true metabolic responses to energy interventions, disease progression, or training adaptations, providing real-time, personalized basis for strategy adjustment. Currently, commercial devices integrating respiratory masks and gas analysis sensors are available, allowing subjects to undergo measurements in free-living conditions, which significantly enhances the frequency of data collection and ecological validity (148). Although devices like smartwatches cannot directly measure REE, they can continuously collect physiological and behavioral parameters such as heart rate, heart rate variability, skin temperature, and physical activity levels (149). By utilizing a small number of individual REE measurements as calibration points, integrating continuously collected wearable device data from the same period, and incorporating comprehensive assessments of patient health records, clinical diagnoses, and laboratory test indicators, machine learning can be used to build a personalized REE prediction model for the patient (148). This AI model can automatically learn the patient’s cardiorespiratory-metabolic relationship, thereby enabling highly accurate and continuous estimation of REE without the need for frequent calorimetric measurements. By integrating multi-dimensional health data, this approach achieves truly personalized, dynamic, and continuous monitoring, providing a more comprehensive basis for clinical decision making.

### 5.3 Monitoring REE in assessing disease progression in patients

The REE of patients with CKD exhibit significant dynamic variations across different stages. In the early stages of CKD, specifically stages 1 to 3, patients do not display noteworthy increases in REE. Research indicates that, despite the presence of metabolic acidosis, insulin resistance, and inflammation, renal compensatory mechanisms are partially preserved, thereby preventing marked elevations in REE (28). As CKD advances to later stages, REE fluctuations become correlated with changes in nutritional status and physical activity levels. By the ESRD phase, REE increases concurrently with a decline in eGFR (129, 150, 151). The studies demonstrated that as the disease progressed toward ESRD, significant alterations in energy expenditure occurred, which, combined with reductions in dietary intake, contributed to an energy imbalance. This imbalance ultimately jeopardized nutritional status and heightened the risk of morbidity and mortality among the affected individuals (137). Consequently, the dynamic alterations in REE can serve as valuable indicators for assessing disease progression in patients. However, research exploring the specific impacts of various types and stages of kidney diseases on REE remains limited.

### 5.4 Interventions through medications or diet to regulate REE

In the context of pharmacological interventions, certain specific medications, such as glucocorticoids, immunosuppressants, diuretics, ACEI, and SGLT2i, may indirectly regulate REE by affecting fluid balance, hormone secretion, or muscle mass during the management of kidney disorders. In future treatments, the careful selection of appropriate medications could be considered in order to maintain the body’s energy balance and stabilize the REE of patients with renal diseases. During dietary management of renal diseases, nutritional therapeutic strategies such as low-protein diets and salt-restricted diets will directly impact nutrient intake and utilization, and may also influence REE by altering body composition. Therefore, by assessing dynamic REE and formulating personalized dietary plans, patients with renal diseases can maintain an appropriate level of REE, thereby promoting the rehabilitation process.

## 6 Conclusion

For patients with kidney diseases, the assessment of the stability and accuracy of REE is of critical significance for formulating nutritional support and therapeutic strategies. Accurately evaluating REE is of great clinical value in preventing and treating complications arising from energy imbalances in kidney disease patients, such as cardiovascular diseases, inflammation, and malnutrition. In the future, research on REE in kidney diseases still needs to be further deepened. On one hand, there is a need to develop more precise and convenient REE measurement technologies to meet clinical practice demands. On the other hand, it is essential to explore the intrinsic relationship between REE and the progression of kidney diseases, as well as the impact of REE changes on the complications of kidney diseases. Additionally, studies should investigate the patterns of REE variation across different types of kidney diseases, at different stages of disease, and under different treatment strategies, thereby providing a robust basis for formulating more precise treatment plans.
